# Neuroplasticity-based novel brain stimulation support intervention options for autistic population

**DOI:** 10.3389/fnhum.2025.1522718

**Published:** 2025-02-14

**Authors:** Pushpal Desarkar

**Affiliations:** ^1^Azrieli Adult Neurodevelopmental Centre, Centre for Addiction and Mental Health, Toronto, ON, Canada; ^2^Campbell Family Mental Health Research Institute, Centre for Addiction and Mental Health, Toronto, ON, Canada; ^3^Department of Psychiatry, University of Toronto, Toronto, ON, Canada

**Keywords:** autism, neuroplasticity, brain stimulation, transcranial magnetic stimulation, electroencephalography

## Abstract

Transcranial magnetic stimulation (TMS), introduced in 1985, has become a vital tool for investigating brain-behaviour relationships and therapeutic interventions. Repetitive TMS (rTMS) as a therapeutic tool has shown promise for various neuropsychiatric conditions, including autism, which affects approximately 1% of the global population. Evidence suggests that atypical neuroplasticity characterizes the neurobiology of autism. Recent studies using TMS paradigms like theta-burst stimulation (TBS) indicate an excessive neuroplasticity or hyper-plasticity in the form of an excessive long-term potentiation (LTP) in the motor cortex of autistic adults compared to neurotypical controls. Hyper-plasticity may negatively impact cognitive and behavioural outcomes. Our proposed neuroplasticity-based rTMS intervention protocols aim to address motor function, sensory sensitivities, and executive function difficulties in autistic adults. We present a testable framework to evaluate neuroplasticity in the motor, sensory, and dorsolateral prefrontal cortices, hypothesizing the presence of hyper-plasticity in autistic adults. We anticipate that this hyper-plasticity underpins motor, sensory, and executive function difficulties in autistic adults. Additionally, we propose investigating the efficacy of bilateral rTMS to reduce hyper-plasticity and improve these functions in autistic adults. This approach not only seeks to enhance therapeutic options but also provides biological insights into the brain mechanisms underlying some of the common autism-associated difficulties.

## Introduction

Since the introduction of transcranial magnetic stimulation (TMS) by Barker et al. (1985), TMS techniques have emerged as promising tools for studying brain-behaviour relationships with unprecedented precision. Repetitive TMS (rTMS) is also an accepted therapeutic intervention now and has received regulatory approval for treating major depressive disorder, migraine with aura, obsessive-compulsive disorder, and nicotine use disorder (Lisanby, 2024). Neuropsychiatric disorders are associated with changes in brain function and structure, which are frequently linked to alterations in neuroplasticity. One rationale for using rTMS as a therapeutic tool is that it affects synaptic and homeostatic neuroplasticity, influencing these mechanisms to potentially improve outcomes (Fitzsimmons et al., 2024). In simple words, neuroplasticity refers to brain’s dynamic ability to modify its structure and function in response to modifying stimuli. Long-term potentiation (LTP) is a well-recognized form of activity-dependent neuroplasticity that leads to a lasting increase in synaptic transmission. In contrast, long-term depression (LTD) is the complementary process that reduces the efficacy of synaptic transmission.

Autism Spectrum Disorder (hereafter referred to as autism) is among the most common neurodevelopmental conditions, affecting roughly 1% of the worldwide population (Zeidan et al., 2022). Despite decades of research, biological mechanisms informing evidence-based support interventions for autistic individuals have remained elusive.

## Uncovering atypical neuroplasticity in autism: insights from TMS research

Converging evidence from genetic (Bourgeron, 2015) to animal models (Markram and Markram, 2010) indicate that the neurobiology of autism is characterized by atypical neuroplasticity. Among animal models of autism, while deficient neuroplasticity was found in some (Uchino et al., 2006; Gilbert and Man, 2017), an excessive neuroplasticity or hyper-plasticity was observed in valproic acid models (Markram and Markram, 2010; Silva et al., 2009). A more direct evidence reflecting hyper-plasticity was consistently observed in the human motor cortex (M1) using TMS (Oberman et al., 2010; Oberman et al., 2012; Oberman et al., 2016; Desarkar et al., 2022) with one exception (Jung et al., 2013). These studies used theta-burst stimulation (TBS) (Huang et al., 2005) and paired associative stimulation (PAS) (Stefan et al., 2000) paradigms, which are well-established neuroplasticity-inducing TMS paradigms. TBS consists of two stimulation protocols: continuous TBS (cTBS) and intermittent TBS (iTBS) (Huang et al., 2005). It is generally believed that iTBS delivered to M1 leads to LTP, which is assessed by the duration of enhancement in motor evoked potentials (MEPs) of the contralateral thumb muscle. In contrast, cTBS protocol is thought to induce LTD, measured by the duration of suppression in MEPs. Of note, there can be significant inter-individual variability in the response to TBS protocols, with some individuals experiencing what can be described as a ‘paradoxical’ response. For instance, cTBS may induce a ‘facilitatory’ (rather than inhibitory) effect in autistic children and adolescents (Jannati et al., 2020). Evidence indicates that various individual factors, such as age, biological sex, gender, handedness, genetics, and the state of neural activation, may all influence the response to TBS protocols (Speranza et al., 2024).

The conventional PAS protocol (Stefan et al., 2000) for M1 consists of the repetitive application of two paired stimuli: (i) electrical stimulation of the right median nerve, followed 25 milliseconds later by (ii) TMS pulse administered to the contralateral M1. This sequence is typically performed with 180 pairs at a frequency of 0.1 Hz over a duration of 30 min. Such stimulation leads to associative LTP-like plasticity in M1.

Our group recently replicated the finding of hyper-plasticity in M1 in autistic adults using TBS (Desarkar et al., 2022). Compared to the age, sex, and intelligence quotient (IQ)-matched neurotypical (NT) adults, LTP-like plasticity was significantly greater among autistic adults. Our finding replicating hyper-plasticity in M1 in autistic adults using TBS is consistent with what was observed in 3 studies (Oberman et al., 2010; Oberman et al., 2012; Oberman et al., 2016). The finding of hyper-plasticity in autistic adults is a direct human translation of the consistent finding of excessive LTP found in the valproic acid animal models of autism (Markram and Markram, 2010; Silva et al., 2009). One explanation of observed hyper-plasticity is the excitation/inhibition imbalance created by over-expression of n-methyl-d-aspartate and/or reduced expression of gamma-aminobutyric acid (GABA) A (GABA-A) or GABA-B receptors observed in the brain of autistic individuals (Rubenstein and Merzenich, 2003). A systematic review of TMS studies found evidence of increased excitation/inhibition ratio in M1 in autism (Masuda et al., 2019).

## Exploring rTMS as a support intervention in autism: a neuroplasticity-based approach

Given the evidence of atypical neuroplasticity, including instances of hyper-plasticity, the use of neuroplasticity-based novel rTMS interventions to support autistic adults presents a promising approach. Such a neuroplasticity-based model of brain stimulation intervention in autism is conceptually based on the premise that hyper-plasticity adversely affects behaviours and cognition. The relationship between hyper-plasticity and behaviour/cognition follows an “inverted U” shape (Pascual-Leone et al., 2011). While deficient neuroplasticity hinders the brain’s ability to adapt to changing conditions, a key ‘insight’ from some animal studies of autism suggest that hyper-plasticity may negatively impact behaviour (Markram and Markram, 2010; Silva et al., 2009). A model outlining a neuroplasticity-pathology continuum proposes that at the circuit level sustained and excessive LTP can lead to excitotoxicity, resulting in neuronal loss and decreased synaptic density, which in turn compromises behaviour (McEachern and Shaw, 1999). In relation to hyper-plasticity in M1, a meta-analysis of post-mortem studies found reduced dendritic spines in M1 among autistic individuals (Fetit et al., 2021).

A recent systematic review identified that interventions improving daily functioning in autistic adults have been identified as one of the top research priorities in the autism community (Roche et al., 2021). Motor function, sensory difficulties, and executive function (EF) difficulties are very common autism-associated difficulties that are considered disabling, as they significantly affect day-to-day functioning (Hedgecock et al., 2018; Travers et al., 2017; Suarez, 2012; Wallace et al., 2016). As a result, they are key therapeutic targets for interventions. However, no quality RCT evidence supports use of clinical intervention to improve any of these there disabling difficulties experienced by autistic adults.

Before developing a neuroplasticity-based model of brain stimulation intervention, there are some key questions and methodological challenges that must be considered and addressed. First, how to assess neuroplasticity using TMS in key regions of interest in the brain in autistic individuals such as frontal cortex, primary sensory cortex (S1), etc.; MEPs are the conventional measure of neuroplasticity in M1 but one key challenge in studying neuroplasticity in other areas of brain such as dorsolateral prefrontal cortex (DLPFC) or S1 is the lack of such peripheral neurophysiological output measure to demonstrate LTP. It was shown that PAS can be combined with electroencephalogram (EEG) (PAS-EEG) to probe neuroplasticity in the DLPFC (Rajji et al., 2013), and S1. Second, what is the association between atypical neuroplasticity and some of the disabling autism-associated difficulties such as EF, sensory, and motor function difficulties? In order for the rTMS intervention to be ‘mechanism-driven’ (i.e., neuroplasticity-based), it is critical to test the strength of the ‘non-linear’ relationship between hyper-plasticity and behaviour/cognition. Third, can rTMS be used to ‘reduce’ hyper-plasticity so that the resulting changes will lead to an improvement in functions? Besides replicating the finding of hyper-plasticity in M1, as a foundation for intervention, our group also collected pilot data using an rTMS protocol designed to strengthen inhibitory mechanisms, which reduced hyper-plasticity in autistic adults (Desarkar et al., 2022). In our pilot study, autistic adults were randomized (1:1) to receive a single session of active or sham rTMS (6,000 pulses at 20 Hz) over M1 and neuroplasticity was reassessed on the next day following rTMS. The mean reduction of LTP (“meanpre—meanpost rTMS’) assessed using TBS indicated a large effect size (partial η^2^ = 0.167) of active rTMS on LTP (Desarkar et al., 2022). In comparison to 1 Hz or 10 Hz rTMS, the 20 Hz rTMS with an extended pulse delivery exhibited a more pronounced inhibitory effect, which reached its peak when a total of 6,000 pulses were administered at this frequency (de Jesus et al., 2014). In the altered excitation/inhibition model of autism (Rubenstein and Merzenich, 2003), hyper-plasticity is likely linked with the increased excitation/inhibition ratio and the reduction of hyper-plasticity in the autism group in our published work by the rTMS could be due to facilitation of inhibition. We had previously published the rationale behind such approach (Desarkar et al., 2015).

## Neuroplasticity-based rTMS support intervention for motor, sensory, and EF difficulties in autism

A novel neuroplasticity-based rTMS support intervention approach for motor, sensory, and EF difficulties in autism are briefly described here. Some of these experiments are ongoing. The targets for these interventions would be M1 (for motor function difficulties), primary sensory cortex (S1) (for atypical tactile sensitivity) and DLPFC (for EF).

Each of these protocols has 3 key objectives and related hypotheses: (a) To assess and compare neuroplasticity in regions of interest (e.g., M1, S1, DLPFC) between autistic adults and NT controls. We anticipate that M1, S1, DLPFC will show hyper-plasticity in autistic adults. (b) To test the association between neuroplasticity and cognition/function (e.g., motor, sensory, and EF). We anticipate that there will be a non-linear incomplete ‘inverted U’ shaped association between neuroplasticity and cognition/function, however, the association will be different for the two groups, i.e., autistic adults will mainly be in the right slope of the inverted U reflecting hyper-plasticity associated with impaired cognition/function, while NT controls will cluster around the centre. (c) To examine the efficacy of bilateral ‘mechanism-driven’ rTMS delivered to M1, S1 or DLPFC in reducing hyper-plasticity and improving motor, sensory, and EF difficulties, respectively, in autistic adults via randomized, double-blind, sham-controlled experiments. We hypothesize that autistic adults receiving active rTMS will have lower plasticity in M1, S1, and DLPFC immediately, and 1 and 4 weeks after the rTMS course compared to autistic adults receiving sham rTMS.

### Neuroplasticity-based rTMS intervention for motor function difficulties delivered to M1

This 5-year study was approved by the Centre for Addiction and Mental Health (CAMH) research ethics board (protocol reference #180-2023), and registered with Clinicaltrials.gov (NCT06497920). In this study, the first step is to assess baseline motor function using standardized tools and M1 neuroplasticity in autistic adults with significant motor function difficulties (*n* = 100) and compare them to NT controls (*n* = 50) matched 2:1 for age, sex, and IQ. All participants are intellectually able adults. Significant motor function difficulties is defined as a standard composite score < 40 (i.e., >1 standard deviation below the mean) on either fine or gross motor composite scores of the Bruininks-Oseretsky Test of Motor Proficiency, Second Edition (Bruininks and Bruininks, 2005). The non-linear incomplete inverted-U association between M1 neuroplasticity and motor function will be investigated among autistic adults and NT controls. The autistic adult participants will then undergo sex-stratified randomization (1:1, double-blind) to receive 5-session of bilateral active or sham rTMS to M1, after which motor function and neuroplasticity in M1 will be assessed immediately, 1-week, and 4-week after the last session of rTMS.

Neuroplasticity in M1 will be assessed using iTBS following our published method^13^. LTP-like plasticity will be assessed by the duration of enhancement in MEPs of the contralateral thumb muscle following iTBS. Active or sham rTMS will be delivered bilaterally to M1. The rTMS paradigm comprises of the delivery of 6,000 pulses (120 trains of 50 pulses with an inter-train interval of 30 s) of active or sham 20 Hz rTMS (Desarkar et al., 2022). rTMS will be delivered at 90% of the resting motor threshold in both conditions (Desarkar et al., 2022).

### Neuroplasticity-based rTMS intervention for EF delivered to DLPFC

Currently, our team is assessing and comparing neuroplasticity in the left DLPFC between autistic adults (*n* = 32) and age, sex, and IQ-matched NT controls (*n* = 32). All participants are right handed and intellectually able. In addition, non-linear incomplete ‘inverted-U’ association between neuroplasticity in the left DLPFC and EF is being investigated among autistic adults and NT controls. The study was approved by the CAMH research ethics board (protocol reference # 135-2019).

Neuroplasticity in the left DLPFC is being assessed using the innovative PAS-EEG method. In the DLPFC PAS-EEG experiment, PAS induced LTP (i.e., PAS-LTP) is defined by potentiation of Cortical Evoked Activity (CEA), which is measured by EEG. Conventional PAS with an inter-stimulus interval (ISI) of 25 millisecond can be used to assess neuroplasticity from DLPFC. PAS-LTP was indexed by PAS-induced significant potentiation of CEA (mean post-PAS/pre-PAS CEA ratio) in the DLPFC (Rajji et al., 2013). We obtained evidence for the feasibility of the PAS-EEG protocol to assess plasticity in the left DLPFC in autistic adults. Our preliminary findings indicated significant hyperplasticity in autistic adults (*n* = 8), compared to NT controls (*n* = 12) (Desarkar et al., 2023). EF is being assessed using a standardised questionnaire and lab-based EF tools.

If hyper-plasticity is identified in the DLPFC and is found to underlie EF difficulties in autistic adults, the previously described ‘mechanism-driven’ rTMS protocol can be employed in a double-blind sham-controlled study. This study would then test the hypothesis that a reduction in neuroplasticity is associated with improvements in EF. The rTMS would be administered bilaterally to DLPFC. The rationale for bilateral delivery of rTMS is based on two key points:

(i) The previous rTMS trial for EF in autistic individuals used bilateral stimulation (Ameis et al., 2020); (ii) Research suggests that DLPFC dominance may align with handedness in some people (Surya et al., 2023). Additionally, because handedness can vary widely among autistic people (Markou et al., 2017), the trial will include autistic participants with left-handed, right-handed, or mixed-handedness.

### Neuroplasticity-based rTMS intervention for sensory difficulties delivered to S1

Our team is also assessing and comparing neuroplasticity in the left S1 between autistic adults (*n* = 32) and age, sex, and IQ-matched NT controls (*n* = 32). All participants are right-handed and intellectually able. The non-linear incomplete ‘inverted-U’ association between neuroplasticity in the S1 and atypical tactile sensitivity is being investigated among autistic adults and NT controls. In addition, our team is also testing if a single session of rTMS protocol described above (Desarkar et al., 2022) can safely reduce hyper-plasticity in S1 in autistic adults. The study was approved by the CAMH research ethics board (protocol reference # 135-2019) (Kariminezhad et al., 2024).

In this study protocol, neuroplasticity in the left S1 of autistic adults and NT control participants is being assessed using a similar PAS-EEG approach, with one adjustment: to generate an LTP-like plasticity, the peripheral stimulation of the median nerve will be preceded by the TMS stimulation of the contralateral S1 by 20 milliseconds. The 20 milliseconds interval is being employed to ensure that the timing of the peripheral nerve stimulation and TMS is aligned with the arrival of the peripheral sensory input in the S1 (Wolters et al., 2005). Atypical tactile sensitivity is being assessed using a standardised questionnaire. Autistic participants are randomized (double-blind, 1:1) to receive a single-session of either sham or active 20 Hz bilateral rTMS over the S1 and then neuroplasticity is re-assessed over the S1 on the same day. The pilot data can be used to design a future randomized double-blind sham-controlled clinical trial to investigate the effectiveness of rTMS in improving atypical tactile sensitivity in autism by reducing S1 hyper-plasticity (Kariminezhad et al., 2024).

## Discussion

Testable neuroplasticity-based brain stimulation interventions aimed at improving motor, sensory, and executive function difficulties in autistic adults are described here.

At this point, there is no known rTMS intervention study targeting sensory or motor function in autism. Previously, Ameis et al. (2020) reported feasibility of the delivery of bilateral (750 pulses/hemisphere, 1,500 pulses in total/session) high-frequency (20 Hz) rTMS intervention to the DLPFC to treat EF difficulties in autistic youth. This study did not find any evidence of the efficacy of either active or sham rTMS to improve EF. However, there are key differences between the Ameis et al. (2020) study and our proposed approach. Our intervention involves a significantly higher number of pulses—6,000 pulses in total/session, compared to 1,500 pulses /session in the Ameis et al. (2020) study. While Ameis et al. (2020) applied the same rTMS protocol that had previously shown promise for improving working memory in schizophrenia (Barr et al., 2013), our approach is ‘mechanism-driven’ in the context of autism, specifically designed to modulate plasticity by addressing the excitation/inhibition imbalance, with the goal of improving EF difficulty in autistic people.

Our proposed approaches have several advantages: (a) Clarifying brain-behaviour relationship in autism: if successful, these studies will identify a brain mechanism, i.e., hyper-plasticity, underlying motor, sensory and EF difficulties in autism; (b) Developing a novel brain stimulation intervention for autism: if successful, our projects will also identify ‘mechanism-driven’ brain stimulation support options to reduce hyper-plasticity in the brain and improve motor, sensory and EF difficulties and thus, outcomes in autism. The estimated lifetime cost of supporting an autistic individual is enormous. Thus, increasing daily functioning and independence will likely have significant cost–benefit that can be tested; (c) Informing future trials: ultimately, this information will provide a foundation to test similar brain stimulation approach for more challenged autistic population subgroups in the future.

## Data Availability

The original contributions presented in the study are included in the article/supplementary material, further inquiries can be directed to the corresponding author.
